# Plasma MCP-1 and Cognitive Decline in Patients with Alzheimer’s Disease and Mild Cognitive Impairment: A Two-year Follow-up Study

**DOI:** 10.1038/s41598-018-19807-y

**Published:** 2018-01-19

**Authors:** Wei-Ju Lee, Yi-Chu Liao, Yen-Feng Wang, I-Feng Lin, Shuu-Jiun Wang, Jong-Ling Fuh

**Affiliations:** 1 Neurological Institute, Taichung Veterans General Hospital, Taichung, Taiwan; 2 Faculty of Medicine, National Yang-Ming University School of Medicine, Taipei, Taiwan; 3 Institute of Clinical Medicine, National Yang-Ming University School of Medicine, Taipei, Taiwan; 4 Department of Neurology, Neurological Institute, Taipei Veterans General Hospital, Taipei, Taiwan; 5 Institute of Public Health, School of Medicine, National Yang-Ming University, Taipei, Taiwan

## Abstract

Monocyte chemoattractant protein-1 (MCP-1, also known as chemokine CCL2) is a vital chemokine that mediates inflammation in Alzheimer’s disease (AD). We analyzed the associations between the baseline plasma MCP-1 level, longitudinal cognitive changes, and genetic effects of CCL2 rs1024611 and its receptor, CC-chemokine receptor 2 (CCR2) rs1799864, in AD. In total, 310 AD patients and 66 mild cognitive impairment (MCI) patients were followed for 2 years, and 120 controls were recruited at baseline for comparison. After adjusting for covariates using one-way analysis of covariance, AD patients had higher plasma MCP-1 levels compared with MCI patients and controls, and severe AD patients had the highest levels. After adjusting for covariates using generalized estimating equation analysis, the results showed that the baseline MCP-1 level was significantly correlated with changes in the two-year Mini-Mental Status Examination (p = 0.046). The A allele of CCR2 rs1799864 was associated with a higher MCP-1 level in AD and MCI patients. In conclusion, plasma MCP-1 might reflect the risk and disease course of AD. A higher plasma MCP-1 level is associated with greater severity and faster cognitive decline. Additionally, the CCR2 polymorphism may play a role in the regulation of MCP-1/CCR2 signaling in AD.

## Introduction

Monocyte chemoattractant protein-1 (MCP-1, also known as chemokine CCL2) and its receptor, CC-chemokine receptor 2 (CCR2), have been implicated in Alzheimer’s disease (AD)^1,2^. Through interactions with CCR2, MCP-1 is the most potent chemokine in the regulation of migration and infiltration of monocytes/macrophages^3^. In AD pathophysiology, MCP-1 is primarily expressed by microglia and macrophages, both of which are involved in the clearance of beta-amyloid (Aβ), myelin degradation, and neuronal loss^2,4^. Clinically, MCP-1 levels in the serum and cerebrospinal fluid (CSF) are elevated in patients with mild cognitive impairment (MCI) and mild AD^5,6^, and there is a significant relationship between CSF and plasma levels of MCP-1^7^. In addition, higher CSF MCP-1 levels have been associated with faster decline in prodromal AD patients^8^. Although a recent systematic review and meta-analysis of biomarkers for the diagnosis of AD^9^ suggested no significant differences when comparing the blood MCP-1 level of AD patients and controls, the sample categories (plasma or serum) and methods of measurement vary in different studies. Further, there have been no reports on the relationship between blood MCP-1 levels and longitudinal cognitive changes in AD and MCI in this meta-analysis.

Genetic studies investigating the relationship between the CCL2 genotype and risks of AD and MCI have reported conflicting results. One study found that the single nucleotide polymorphism (SNP) at the CCL2 promoter (A-2518G, rs1024611) did not correspond with the risk or clinical outcomes of AD^10^. Another study showed that the G allele of CCL2 A-2518G slightly influenced MCI conversion to AD and also showed that CCL2 genotypes did not affect the plasma MCP-1 levels^11^. These results suggest that the MCP-1 levels may not be directly associated with the CCL2 promoter polymorphism. Genetic polymorphisms of CCR2 are alternative targets for investigation.

Our objective is to investigate the role of the plasma MCP-1 levels in AD and MCI patients to obtain a better understanding of its clinical significance, especially on the longitudinal cognitive changes and genetic effects of CCL2 and CCR2.

## Results

### Subjects and demographics

In total, 310 AD patients (171 males/139 females; mean age = 80.1 ± 7.2 years; mean education = 10.1 ± 4.6 years), 66 MCI patients (35 males/31 females; mean age = 75.4 ± 8.2 years; mean education = 10.5 ± 4.9 years), and 120 controls (65 males/55 females; mean age = 74.9 ± 7.8 years; mean education = 10.7 ± 5.3 years) were recruited for the study. AD subjects were older than patients in the MCI and control groups; however, there were no differences in the sex ratio or years of education among the groups. The mean Mini-Mental Status Examination (MMSE) scores of patients with AD, MCI and controls was 18.0 ± 6.0, 25.7 ± 2.9, and 27.6 ± 2.5, respectively. The detailed demographic data are shown in Table 1.

**Table 1:** Demographic and neuropsychiatric data of the study subjects.

	Control (n = 120)	MCI (n = 66)	AD (n = 310)	P value*
Male patients	65 (54.2%)	35 (53.0%)	171 (55.2%)	0.95
Age, year	74.9 (7.8)	75.4 (8.2)	80.1 (7.2)	<0.001
Education years	10.7 (5.1)	10.5 (4.9)	10.1 (4.6)	0.47
BMI	24.1 (2.7)	24.3 (3.9)	23.7 (3.5)	0.27
CCI	4.1 (1.7)	3.9 (1.2)	4.1 (1.1)	0.37
Disease duration, year	—	3.3 (3.0)	4.3 (4.1)	
CDR				
0.5	—	66 (100%)	22 (7.1%)	
1.0	—	—	195 (62.9%)	
2.0	—	—	80 (25.8%)	
3.0	—	—	13 (4.2%)	
APOE ε4 carrier	20 (16.7%)	15 (22.7%)	125 (40.3%)	<0.001
MMSE	27.6 (2.5)	25.7 (2.9)	18.0 (6.0)	<0.001
Delayed recall	—	4.6 (2.7)	1.2 (1.9)	<0.001
Category verbal fluency	—	9.3 (2.6)	6.2 (3.0)	<0.001
Forward digit span	—	9.6 (2.6)	8.4 (3.1)	0.007
Backward digit span	—	5.1 (2.0)	3.9 (2.0)	<0.001
Modified Boston naming	—	13.5 (1.2)	11.2 (3.1)	<0.001

The values indicate means with standard deviations unless otherwise indicated.

Post hoc Tukey test: Age: AD vs. control, p < 0.001; AD vs. MCI, p < 0.001; MMSE: AD vs. control, p < 0.001; AD vs. MCI, p < 0.001; MCI vs. control, p = 0.04.

MCI, mild cognitive impairment; AD, Alzheimer’s disease; BMI, body mass index; CCI, Charlson comorbidity index; CDR, Clinical Dementia Rating; APOE, apolipoprotein E; MMSE, Mini-Mental Status Examination.

^*^Chi-square test, two-sample t-test, Mann-Whitney U test, or one-way analysis of variance.

### Baseline Plasma MCP-1 Levels

The baseline plasma MCP-1 level in AD patients was 245.6 ± 102.8 μg/ml, that in MCI patients was 204.0 ± 67.8 μg/ml and that in controls was 191.3 ± 87.6 μg/ml. Among AD patients, the baseline plasma MCP-1 level in very mild AD patients (Clinical Dementia Rating (CDR) = 0.5) was 232.6 ± 86.7 μg/ml, that in mild AD patients (CDR = 1) was 240.2 ± 99.6 μg/ml, that in moderate AD patients (CDR = 2) was 246.4 ± 97.2 μg/ml, and that in severe AD patients (CDR = 3) was 342.8 ± 157.1 μg/ml. After adjusting for age and the Charlson comorbidity index (CCI), AD patients had higher baseline plasma MCP-1 levels compared with the MCI and control groups and severe AD patients had higher plasma MCP-1 levels compared with mild and moderate AD patients (AD vs. control, p < 0.001; AD vs. MCI, p = 0.02; CDR = 3 vs. CDR = 0.5, p = 0.01; CDR = 3 vs. CDR = 1, p = 0.002; CDR = 3 vs. CDR = 2, p = 0.007) (Tables 2 and 3). There was a significant positive correlation between disease duration and the baseline plasma MCP-1 level in MCI group (γ = 0.27, p = 0.03). However, there was no significant correlation between the MCP-1 level in AD group (γ = −0.004, p = 0.95).

**Table 2:** Results of one-way analysis of covariance (ANCOVA) analyzing the differences in baseline plasma MCP-1 levels between the 3 patient groups.

Parameter	B	SE	t value	P value
Intercept	2.15	0.09	23.24	<0.001
Age	0.002	0.001	1.53	0.13
CCI	0.009	0.008	1.11	0.27
Control	−0.10	0.02	−5.06	<0.001
MCI	−0.06	0.02	−2.34	0.02
AD	Ref			

Dependent variable: plasma MCP-1 level (log transformation).

MCP-1, Monocyte chemoattractant protein-1; CCI, Charlson Comorbidity Index; MCI, mild cognitive impairment; AD, Alzheimer’s disease; SE, standard error.

**Table 3:** Results of one-way analysis of covariance (ANCOVA) analyzing the differences in baseline plasma MCP-1 levels in AD patients.

Parameter	B	SE	t value	P value
Intercept	2.25	0.13	17.08	<0.001
Age	0.002	0.002	1.26	0.21
CCI	0.02	0.01	1.15	0.25
CDR = 0.5	−0.15	0.06	−2.55	0.01
CDR = 1	−0.15	0.05	−3.01	0.002
CDR = 2	−0.14	0.05	−2.73	0.007
CDR = 3	Ref			

Dependent variable: plasma MCP-1 level (log transformation).

MCP-1, Monocyte chemoattractant protein-1; CCI, Charlson Comorbidity Index; AD, Alzheimer’s disease; CDR, clinical dementia rating; SE, standard error.

### Associations Between the Baseline MCP-1 Levels and Longitudinal Cognitive Decline

In total, 285 AD and MCI patients (80.5%) completed the one-year follow-up (22 patients died between the baseline and 1-year follow-up) and 245 patients (71.0%) completed the two-year follow-up (9 patients died between the 1-year and 2-year follow-up). First, we divided the AD and MCI patients who had longitudinal follow-up data into the categories of cognitive decliner and cognitive non-decliner according to the changes in the MMSE scores: cognitive decliner (1-year MMSE decrease ≥ 2) and cognitive non-decliner (1-year MMSE decrease < 2)^12^. Using logistic regression adjusting for age, apolipoprotein E (APOE) ε4 carrier status, sex, years of education, and CCI, the MCP-1 level for the cognitive decliner group was higher than that for the cognitive non-decliner group (p = 0.02) (Supplementary Table 1). Second, Table 4 shows the generalized estimating equation (GEE) analysis of two-year MMSE changes adjusted for age, years of education, gender, time, APOE ε4 carrier status, baseline MMSE scores, and CCI. The GEE analysis revealed that the baseline MCP-1 level was significantly correlated with the 2-year MMSE changes (B = −1.75, 95% CI = −3.47 to −0.03, p = 0.046).

**Table 4:** Results of the generalized estimating equation analyzing the effect of 2-year MMSE changes in patients with AD and MCI.

	Β	SE	95% CI	P value
Gender				
Male	−0.03	0.27	(−0.56, 0.51)	0.93
Female	Ref			
MCP-1 level	−1.75	0.88	(−3.47, −0.03)	0.046
APOE ε4				
non-carrier	0.47	0.32	(−0.16, 1.10)	0.14
carrier	Ref			
Age	0.04	0.02	(−0.01, 0.08)	0.12
Years of education	−0.04	0.03	(−0.10, 0.03)	0.25
Time	−1.19	0.14	(−1.46, −0.92)	< 0.001
Baseline MMSE	0.96	0.03	(0.91, 1.00)	< 0.001
CCI	−0.22	0.16	(−0.53, 0.10)	0.19

Adjusted for age, years of education, gender, time, APOE ε4 carrier, and baseline MMSE.

MMSE, Mini-Mental Status Examination; AD, Alzheimer’s disease; MCI, mild cognitive impairment; MCP-1, Monocyte chemoattractant protein-1; APOE, apolipoprotein E; CCI, Charlson comorbidity index.

### The Genetic Effects of the CCL2 and CCR2 Genotypes on the Plasma MCP-1 Levels

Table 5 shows the distribution of CCL2 and CCR2 genotypes in AD patients, MCI patients, and control subjects. The genotype distributions of CCL2 rs1024611 and CCR2 rs1799864 were in Hardy-Weinberg equilibrium (p = 0.1 and 0.46, respectively). The genotype distributions of these two SNPs were similar among the three patient groups (AD, MCI, and controls), suggesting no association between the CCR2/CCL2 polymorphisms and AD/MCI risk.

**Table 5:** The distribution of the CCL2 and CCR2 genotypes in AD patients, MCI patients, and control subjects.

	Control (n = 120)			MCI (n = 66)			AD (n = 310)			AD and MCI (n = 376)		
	GG	AG	AA	GG	AG	AA	GG	AG	AA	GG	AG	AA
CCL2	39	53	28	22	36	8	84	163	63	106	199	71
rs1024611	(32.5%)	(44.2%)	(23.3%)	(33.3%)	(54.5%)	(12.1%)	(27.1%)	(52.6%)	(20.3%)	(28.2%)	(52.9%)	(18.9%)
Additive model	Ref			p = 0.26, adjusted p = 0.30*			p = 0.73, adjusted p = 0.71*			p = 1.00, adjusted p = 0.74*		
CCR2	75	40	5	42	22	2	185	108	17	227	130	19
rs1799864	(62.5%)	(33.3%)	(4.2%)	(63.6%)	(33.3%)	(3.0%)	(59.7%)	(34.8%)	(5.5%)	(60.4%)	(34.6%)	(5.1%)
Additive model	Ref			p = 0.75, adjusted p = 0.77*			p = 0.51, adjusted p = 0.69*			p = 0.62, adjusted p = 0.84*		

MCI, mild cognitive impairment; AD, Alzheimer’s disease; CCL2, Chemokine ligand 2; CCR2, CC-chemokine receptor 2.

*Multivariate logistic regression model adjusting age, gender, and APOE ε4 carrier status.

**Figure 1:**
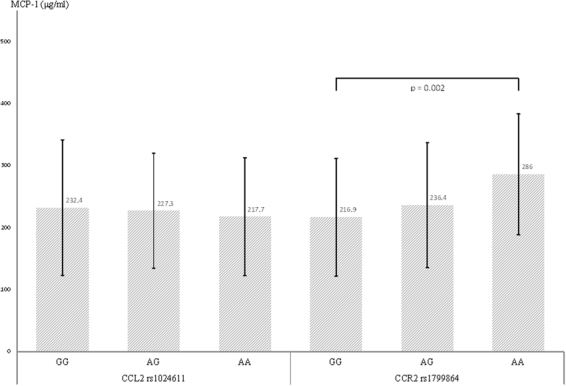
The effects of the CCL2 and CCR2 genotypes on the plasma MCP-1 levels. Plasma MCP-1 levels were similar among the different genotypes of CCL2 rs1024611 in all groups of participants. However, the A allele of CCR2 rs1799864 appeared to exert an additive effect on the plasma MCP-1 levels, with the highest levels observed in the AA genotype, followed by the AG genotype, and the lowest levels were observed in the GG genotype. The p-value was calculated by multiple linear regression models while adjusting for age, sex, APOE ε4 carrier status, and CCI. The error bars indicate standard deviation. Abbreviations: CCL2, Chemokine ligand 2; CCR2, CC-chemokine receptor 2; MCP-1, Monocyte chemotactic protein-1; APOE, apolipoprotein E; CCI, Charlson comorbidity index.

The average MCP-1 levels were similar among the different genotypes of CCL2 rs1024611 in all groups of participants (Fig. 1, left side). However, the A allele of CCR2 rs1799864 appeared to exert an additive effect on the plasma MCP-1 levels, with the highest levels observed in the AA genotype, followed by the AG genotype, and the lowest levels were observed in the GG genotype (p < 0.001; adjusted p < 0.002, Fig. 1, right side). Table 6 shows the relationship between the genotypes and plasma MCP-1 levels in each patient group. The association between the MCP-1 level and CCR2 rs1799864 was significant in AD patients (p = 0.01; adjusted p = 0.01) and MCI patients (p = 0.001, adjusted p = 0.003), and there was no significant association between the MCP-1 level and CCR2 rs1799864 in controls. In addition, there was no association between the MCP-1 level and CCL2 rs1024611 within each patient subgroup.

**Table 6:** The relationship between the CCL2 and CCR2 genotypes and plasma MCP-1 levels in AD patients, MCI patients, and control subjects.

CCL2 rs1024611	Control (n = 120)			MCI (n = 66)			AD (n = 310)			AD and MCI (n = 376)		
	GG	AG	AA	GG	AG	AA	GG	AG	AA	GG	AG	AA
MCP-1 levels	220.8 ± 118.7	173.5 ± 67.5	183.8 ± 57.3	207.5 ± 76.7	211.9 ± 66.2	158.8 ± 20.5	244.3 ± 111.1	248.2 ± 97.4	240.3 ± 106.4	236.7 ± 105.7	241.7 ± 93.5	231.1 ± 103.7
Additive model	p = 0.19, adjusted p = 0.30*			p = 0.32, adjusted p = 0.41*			p = 0.99, adjusted p = 0.81*			p = 0.94, adjusted p = 0.80*		
CCR2 rs1799864	GG	AG	AA	GG	AG	AA	GG	AG	AA	GG	AG	AA
MCP-1 levels	182.6 ± 83.7	209.6 ± 96.5	174.3 ± 51.4	189.3 ± 71.2	222.5 ± 46.2	310.5 ± 82.7	237.0 ± 98.6	249.1 ± 108.4	316.0 ± 87.3	228.2 ± 95.8	244.6 ± 100.9	315.4 ± 84.6
Additive model	p = 0.23, adjusted p = 0.30*			p = 0.001, adjusted p = 0.003*			p = 0.01, adjusted p = 0.01*			p < 0.001, adjusted p = 0.001*		

The values indicate means with standard deviations.

MCI, mild cognitive impairment; AD, Alzheimer’s disease; CCL2, Chemokine ligand 2; CCR2, CC-chemokine receptor 2; MCP-1, Monocyte chemoattractant protein-1.

*Multivariate linear regression model adjusting age, gender, APOE ε4 carrier status, and Charlson comorbidity index.

### Gene-gene Interaction in Relation to the MCP-1 Level or AD and MCI Risks

We first used the logistic regression model of generalized multifactor dimensionality reduction (GMDR) to examine gene-gene interactions among any combination of the three loci, CCL2 rs1024611, CCR2 rs1799864 and APOE ε4 carrier status. None of the best combinations of interacting loci chosen by the GMDR software achieved statistical significance (Supplementary Table 2). We then studied the association between the plasma MCP-1 levels and gene-gene interactions in AD and MCI patients. Neither the gene-gene interaction between CCR2 and CCL2 nor the interaction among CCR2, CCL2 and APOE ε4 was a significant predictor of plasma MCP-1 levels (Supplementary Table 3).

## Discussion

The current study demonstrates that plasma MCP-1 levels are higher in AD patients than in MCI patients and controls. In addition, plasma MCP-1 levels increase with the increasing severity of AD. Furthermore, higher plasma MCP-1 levels are associated with faster cognitive decline in AD and MCI patients at the two-year follow-up, and AD and MCI patients with cognitive decline had higher baseline plasma MCP-1 levels. These results suggest that plasma MCP-1 levels are associated with the risk and disease course of AD and might be used as a prognostic biomarker for the rate of cognitive decline of AD and MCI patients.

Previous studies have shown a higher plasma MCP-1 level in MCI and AD patients compared to healthy controls^5,13^. Significantly increased MCP-1 levels were reported in MCI and mild AD but not severe AD compared with controls^5^. However, according to our results, the plasma MCP-1 levels are significantly increased in AD patients compared with MCI patients and controls, and the levels are significantly increased in severe AD (CDR = 3) compared with minor stages of AD. The increased plasma MCP-1 levels observed in the late clinical AD stages might suggest that MCP-1 upregulation is an ongoing process from mild to severe AD.

MCP-1 is produced by many cell types, including endothelial cell, fibroblast, epithelial cell, smooth muscle cell, mesangial cell, astrocyte, monocyte, and microglia. Among these cell types, monocytes/macrophages are found to be the major source of MCP-1. The main function of MCP-1 is regulation of the migration and infiltration of monocytes, memory T lymphocytes, and natural killer cells^14^. In AD, recent evidence suggests that the MCP-1 related inflammatory process is associated with Aβ and microglia accumulations^15–17^ and the MCP-1 levels are increased in the CSF and in brain tissue of AD patients^6,18^. Another study demonstrated systemic immune system alterations in AD with decreased expression of monocyte CCR2 and increased plasma MCP-1^13^. An animal study using a transgenic mouse model of AD (Tg2576) showed that CCR2 deficiency accelerates early disease progression and markedly impairs microglial accumulation. Early microglial accumulation in AD is CCR2 dependent and is capable of inducing microglial accumulation, probably through recruitment of mononuclear phagocytes from the blood and bone marrow^15^. These findings suggest that peripheral immunologic alterations may be associated with neuroinflammation in AD and MCP-1 in peripheral blood may attract blood-derived monocytes to migrate into brain^19^. The possible interaction between peripheral inflammation and neuroinflammation may provide the opportunity for using blood-based biomarkers to monitor the changes in neuroinflammation^13^. The involvement of cytokines and chemokines in AD is evident from studies in which their concentrations in both peripheral blood and CSF are elevated^7,20^. Among these molecules, MCP-1 has a significant relationship between the CSF and plasma levels^7^. Therefore, the possible usefulness of plasma MCP-1 to reflect the severity of neuroinflammation can be considered.

In the present study, plasma MCP-1 levels positively correlate with the disease duration during the stage of MCI, but not during the stages of AD. The lack of a consistent relationship between the MCP-1 level and disease duration during different disease stages (MCI and AD) might suggest alterations in plasma MCP-1 levels during different disease stages. This conclusion is supported by a hypothetical model of dynamic biomarkers, including neuroinflammation in AD^21^, which suggests that microglial activation does not increase steadily form early to late stages of the disease process. In our 2-year longitudinal study, the high baseline MCP-1 level is associated with the rate of cognitive decline in AD and MCI patients and may indicate the deleterious effects of MCP-1 during AD and MCI as well as suggest that plasma MCP-1 levels may be useful for monitoring disease progression.

There were no significant differences in the genotype distributions of CCL2 rs1024611 or CCR2 rs1024611 among participants. These results agree with previous studies^10,11^. The current study found no association between the CCL2 rs1024611 genotypes and plasma MCP-1 levels among participants; however, the CCR2 rs1799864 genotype was associated with the plasma MCP-1 levels in AD and MCI, but not in controls. These results suggest that the stability or expression of CCR2 may play an important role in AD and that high plasma MCP-1 levels may be a response to altered expression or signaling of CCR2 in AD pathogenesis. However, because the genotype distributions are not different between diagnostic groups, mRNA expression of CCR2 as well as CCR2 expression in circulating monocytes should be further investigated to support this finding. In a study on HIV infection, the CCR2-V64I polymorphism was found to affect the stability of the CCR2A isoform^22^. Another study using small interfering RNA that led to functional inhibition of CCR2 showed significant down-regulation of CCL2 mRNA levels^23^. These findings further support the theory that the CCR2 gene may play a role in the regulation of MCP-1/CCR2 signaling. Interestingly, pharmaceuticals and antibodies that modify MCP-1/CCR2 signaling have already been developed^24,25^ and may be considered for therapeutic trials in prodromal AD. Our results suggest that CCR2 could be a primary target for treatment rather than MCP-1 itself. However, further studies on the MCP-1/CCR2 signaling regulatory pathways in AD should be performed.

The current study has some limitations and weaknesses. First, the diagnoses of AD and MCI were made using only clinical criteria without biomarkers of Aβ deposition and tau-mediated neuronal degeneration, which may influence the diagnostic accuracy. We cannot analyze the relationships between biomarkers of Aβ deposition, tau-mediated neuronal degeneration and MCP-1 levels. Second, the mean age of AD patients was older than that of MCI patients and controls, which may impact the results of the genetic effects on disease as well as plasma MCP-1 levels among different groups. However, after adjusting for age, AD patients still had higher baseline plasma MCP-1 levels compared with the MCI and control groups, and the genetic effects of CCR2 on the plasma MCP-1 level in patients with AD and MCI were still statistically significant. Third, we used the MMSE to evaluate longitudinal cognitive changes, which might underestimate cognitive decline. Serial brain imaging studies should be performed in the future to reinforce our findings. Fourth, the lack of a longitudinal follow-up in the control participants is another limitation.

In conclusion, our findings describe the association between plasma MCP-1 levels and the clinical stages of AD. A higher plasma MCP-1 level is associated with greater severity and faster cognitive decline. In addition, the CCR2 polymorphism may play a more important role than the CCL2 polymorphism in modulating the MCP-1 level in AD. These results support the need for future investigations of drugs that can modify MCP-1/CCR2 signaling and highlight the importance of early initiation of anti-neuroinflammatory treatments.

## Methods

### Participants

Normal healthy controls, MCI patients and AD patients were recruited from the outpatient clinics of the Taipei Veterans General Hospital and Taichung Veterans General Hospital in Taiwan during the period from August 2012 to June 2014. Healthy controls were volunteers with normal cognitive function who were recruited from outpatient clinics at the two hospitals. An AD diagnosis was made during a multidisciplinary consensus meeting according to the clinical criteria for probable AD as described by the National Institute on Aging–Alzheimer’s Association^26^. A diagnosis of MCI was made according to the revised consensus criteria from 2004^27^. When the cognitive symptoms of patients significantly interfered with their abilities or work functions, which were evaluated through a combination of history-taking and cognitive assessment, they were diagnosed with dementia. The cut-off value for diagnosis of MCI was set at 1.5 standard deviations below the age-adjusted norm for the logical memory test of the Wechsler Memory Scale III^28^. Other inclusion criteria included an age at onset greater than 60 years and the availability of a caregiver who could provide collateral patient history. All patients received a standardized evaluation that included a clinical interview, neuropsychological assessment, laboratory tests and brain magnetic resonance imaging. The exclusion criteria were subjects who had significant neurological diseases other than AD that may affect cognition, including Parkinson’s disease, vascular dementia, normal pressure hydrocephalus, brain tumor, progressive supranuclear palsy, seizure disorder, subdural hematoma, multiple sclerosis, or a history of significant head trauma followed by persistent neurologic defaults or known structural brain abnormalities. Subjects taking anti-inflammatory or immunosuppressive drugs were also excluded. The disease duration was defined as the period between the initial onset of symptoms reported by the caregiver and participation in the study. This research project was approved by the institutional review boards at Taipei Veterans General Hospital (IRB number 2012-05-033B) and Taichung Veterans General Hospital (IRB number SF12171). All methods were performed in accordance with the relevant guidelines and regulations. Informed consent was obtained from all patients and their caregivers before study participation.

### Clinical evaluation and procedures

Cognitive function was assessed by standard procedures at baseline. The MMSE^29^ was used to assess global cognition. The CDR^30^ was administered to determine the severity of dementia. In this study, we used the CDR as a severity rating scale rather than a diagnostic scale. Therefore, subjects with a CDR of 0.5 may meet the criteria stated above for MCI or they may represent very mild AD^31,32^. Additionally, the 12-item memory test, modified 15-item Boston Naming Test, category verbal fluency test, and forward and backward digit span test were used to assess short-term memory, language, executive function, attention and working memory, respectively. The CCI was used to represent the comorbidities of patients and healthy controls. Longitudinal follow-up was performed in MCI and AD patients at one-year intervals for two years. At the yearly follow-up, the MMSE and the CDR were administered again.

### DNA analysis

Genomic DNA was isolated from whole blood using a Gentra Puregene kit according to the manufacturer’s protocol (Qiagen, Hilden, Germany). The presence of the ε2, ε3, and ε4 alleles of the APOE gene were determined by genotyping of SNPs rs429358 and rs7412. The APOE ε4 carrier was defined as having at least one ε4 allele (including ε2/ε4, ε3ε4, and ε4/ε4). The promoter SNP rs1024611 (A-2518G) in CCL2 and non-synonymous SNP rs1799864 (V64I) in CCR2 were investigated because both SNPs were previously shown to have functional impacts on genetic expression^10,33^. Genotyping of rs429358, rs7412, rs1024611 and rs1799864 was performed using the TaqMan genotyping assay (Applied Biosystems, Foster City, CA, USA). Polymerase chain reactions were performed in 96-well microplates with an ABI 7500 real-time PCR machine (Applied Biosystems). Allele discrimination was achieved by detecting fluorescence using System SDS software version 1.2.3 (Applied Biosystems).

### Plasma MCP-1 levels measurement

Freshly drawn venous blood was collected at baseline in tubes containing EDTA, which were then centrifuged, and the samples were stored in polypropylene tubes at -80 °C until biochemical analysis. Plasma was used for analysis because it provides the most accurate cytokine measurements^34^. Plasma MCP-1 levels were measured in duplicate using MILLIPLEX^®^ MAP kits (bead-based) following the manufacturer’s instructions and standard procedures. The sample storage time was 3.1 ± 1.84 months. The coefficient of variance for each sample was less than 20%.

### Statistical analysis

The skewness and kurtosis of continuous variables, including age, years of education, body mass index (BMI), MMSE scores, category verbal fluency test scores, and forward and backward digit span scores, were between –1 and 1. However, the skewness value of delayed recall of the 12-item memory test, modified 15-item Boston Naming Test, and plasma MCP-1 measurement exceeded 1. Thus, we log transformed the plasma MCP-1 values for further analysis^35^. The Chi-square test, independent two-sample t-test, Mann-Whitney U test, and one-way analysis of variance with the post-hoc Tukey test were used to examine the association between demographic and neuropsychiatric variables in controls and patients with AD and MCI when appropriate. One-way analysis of covariance was used to examine the differences in baseline plasma MCP-1 levels between controls and patients with AD and MCI after adjusting for age and the CCI. Pearson correlation analysis was used to assess the relationship between plasma MCP-1 levels and disease duration. We divided the AD and MCI patients who had longitudinal follow-up data into the categories of cognitive decliner and cognitive non-decliner according to the changes in the MMSE scores: cognitive decliner (1-year MMSE decrease ≥ 2) and cognitive non-decliner (1-year MMSE decrease < 2)^12^. A logistic regression with adjustments for age, APOE ε4 carrier status, sex, years of education, and CCI was used to analyze the relationship between the MCP-1 level and cognitive decliner or cognitive non-decliner groups. The GEE method was used to analyze the correlation between the baseline plasma MCP-1 level and longitudinal MMSE changes occurring at the two-year follow-up, adjusting for age, years of education, gender, time, baseline MMSE, APOE ε4 carrier status, and CCI. For the GEE analysis, we used a robust estimator for the covariance matrix and selected autoregressive (1) for the working correlation matrix. Estimated β-values with 95% confidence intervals (CI) were calculated. Statistical significance was defined as p < 0.05.

Hardy-Weinberg equilibrium was tested using the goodness-of-fit test. A multivariate logistic regression analysis was used to compare the genotype distributions between AD patients, MCI patients, and controls and to evaluate the genetic effects of the CCL2 promoter and CCR2 non-synonymous SNPs (rs1024611 and rs1799864) while adjusting for age, sex, and APOE ε4 carrier status. Lastly, the relationships between the plasma MCP-1 levels and CCL2 and CCR2 genotypes were also analyzed using multiple linear regression models while adjusting for age, sex, APOE ε4 carrier status, and the CCI separately in AD patients, MCI patients, and controls. All statistical analyses were performed with SPSS software, version 18.0 (IBM, Inc., Armonk, NY, USA) and SAS statistical software, version 9.4, with p < 0.05 used to indicate statistical significance.

Gene-gene interactions among CCL2 rs1024611, CCR2 rs1799864 and APOE ε4 carrier statuses were investigated using the GMDR software (http://www.ssg.uab.edu/gmdr/)^36^, which is a method to reduce the dimensionality of multilocus information and improve the identification of polymorphism combinations associated with disease risk. The best candidate interaction model was selected across all of the multilocus models that maximized the testing accuracy and cross-validation consistency. A permutation with 1000 replications was employed to determine the accuracy of the p-value statistics in GMDR, and an alpha level of 0.05 was considered statistically significant.

### Data availability

The datasets analyzed during the current study are available from the corresponding author upon reasonable request.

## Electronic supplementary material

Supplementary Table 1,2,3

## References

[1] Conductier G, Blondeau N, Guyon A, Nahon JL, Rovere C (2010). The role of monocyte chemoattractant protein MCP1/CCL2 in neuroinflammatory diseases. Journal of neuroimmunology.

[2] Banisadr G (2005). Constitutive neuronal expression of CCR2 chemokine receptor and its colocalization with neurotransmitters in normal rat brain: functional effect of MCP-1/CCL2 on calcium mobilization in primary cultured neurons. The Journal of comparative neurology.

[3] Sozzani S (1994). Receptors and transduction pathways for monocyte chemotactic protein-2 and monocyte chemotactic protein-3. Similarities and differences with MCP-1. Journal of immunology (Baltimore, Md. 1950).

[4] Britschgi M, Wyss-Coray T (2007). Systemic and acquired immune responses in Alzheimer’s disease. International review of neurobiology.

[5] Galimberti D (2006). Serum MCP-1 levels are increased in mild cognitive impairment and mild Alzheimer’s disease. Neurobiology of aging.

[6] Galimberti D (2006). Intrathecal chemokine synthesis in mild cognitive impairment and Alzheimer disease. Archives of neurology.

[7] Sun, Y. X. *et al*. Inflammatory markers in matched plasma and cerebrospinal fluid from patients with Alzheimer’s disease. *Dementia and geriatric cognitive disorders* **16**, 136–144, doi:71001 (2003). 10.1159/000071001 12826739

[8] Westin K (2012). CCL2 is associated with a faster rate of cognitive decline during early stages of Alzheimer’s disease. PloS one.

[9] Olsson B (2016). CSF and blood biomarkers for the diagnosis of Alzheimer’s disease: a systematic review and meta-analysis. The Lancet. Neurology.

[10] Huerta C (2004). Chemokines (RANTES and MCP-1) and chemokine-receptors (CCR2 and CCR5) gene polymorphisms in Alzheimer’s and Parkinson’s disease. Neuroscience letters.

[11] Porcellini E, Ianni M, Carbone I, Franceschi M, Licastro F (2013). Monocyte chemoattractant protein-1 promoter polymorphism and plasma levels in alzheimer’s disease. Immunity & ageing: I & A.

[12] Morris JC (1993). The consortium to establish a registry for Alzheimer’s disease (CERAD). Part IV. Rates of cognitive change in the longitudinal assessment of probable Alzheimer’s disease. Neurology.

[13] Zhang R (2013). Systemic immune system alterations in early stages of Alzheimer’s disease. Journal of neuroimmunology.

[14] Deshmane SL, Kremlev S, Amini S, Sawaya BE (2009). Monocyte chemoattractant protein-1 (MCP-1): an overview. Journal of interferon & cytokine research: the official journal of the International Society for Interferon and Cytokine Research.

[15] El Khoury J (2007). Ccr2 deficiency impairs microglial accumulation and accelerates progression of Alzheimer-like disease. Nature medicine.

[16] Naert G, Rivest S (2011). CC chemokine receptor 2 deficiency aggravates cognitive impairments and amyloid pathology in a transgenic mouse model of Alzheimer’s disease. The Journal of neuroscience: the official journal of the Society for Neuroscience.

[17] Kiyota T (2013). CCL2 affects beta-amyloidosis and progressive neurocognitive dysfunction in a mouse model of Alzheimer’s disease. Neurobiology of aging.

[18] Sokolova A (2009). Monocyte chemoattractant protein-1 plays a dominant role in the chronic inflammation observed in Alzheimer’s disease. Brain pathology (Zurich, Switzerland).

[19] Britschgi M, Wyss-Coray T (2007). Immune cells may fend off Alzheimer disease. Nature medicine.

[20] Teunissen CE, de Vente J, Steinbusch HW, De Bruijn C (2002). Biochemical markers related to Alzheimer’s dementia in serum and cerebrospinal fluid. Neurobiology of aging.

[21] Calsolaro V, Edison P (2016). Neuroinflammation in Alzheimer’s disease: Current evidence and future directions. Alzheimers Dement.

[22] Nakayama EE, Tanaka Y, Nagai Y, Iwamoto A, Shioda T (2004). A CCR2-V64I polymorphism affects stability of CCR2A isoform. AIDS (London, England).

[23] Begin-Lavallee, V. *et al*. Functional inhibition of chemokine receptor CCR2 by dicer-substrate-siRNA prevents pain development. *Molecular pain* **12**10.1177/1744806916653969 (2016). 10.1177/1744806916653969 PMC4956154 27306408

[24] Kashyap S (2016). Blockade of CCR2 reduces macrophage influx and development of chronic renal damage in murine renovascular hypertension. American journal of physiology. Renal physiology.

[25] Brana I (2015). Carlumab, an anti-C-C chemokine ligand 2 monoclonal antibody, in combination with four chemotherapy regimens for the treatment of patients with solid tumors: an open-label, multicenter phase 1b study. Targeted oncology.

[26] McKhann GM (2011). The diagnosis of dementia due to Alzheimer’s disease: recommendations from the National Institute on Aging-Alzheimer’s Association workgroups on diagnostic guidelines for Alzheimer’s disease. Alzheimers Dement.

[27] Winblad B (2004). Mild cognitive impairment–beyond controversies, towards a consensus: report of the International Working Group on Mild Cognitive Impairment. Journal of internal medicine.

[28] Wechsler D Wechsler Memory Scale-III. San Antonio, Texas: The Psychological Corporation (1998)

[29] Folstein MF, Folstein SE, McHugh PR (1975). “Mini-mental state”. A practical method for grading the cognitive state of patients for the clinician. Journal of psychiatric research.

[30] Morris JC (1993). The Clinical Dementia Rating (CDR): current version and scoring rules. Neurology.

[31] Petersen RC (2004). Mild cognitive impairment as a diagnostic entity. Journal of internal medicine.

[32] Morris JC (2001). Mild cognitive impairment represents early-stage Alzheimer disease. Archives of neurology.

[33] Rovin BH, Lu L, Saxena R (1999). A novel polymorphism in the MCP-1 gene regulatory region that influences MCP-1 expression. Biochemical and biophysical research communications.

[34] de Jager W, Bourcier K, Rijkers GT, Prakken BJ, Seyfert-Margolis V (2009). Prerequisites for cytokine measurements in clinical trials with multiplex immunoassays. BMC immunology.

[35] Munro, B. H. Statistical Methods for Health Care Research. 4th edn, 42–46 (Lippincott, 2001).

[36] Lou XY (2007). A generalized combinatorial approach for detecting gene-by-gene and gene-by-environment interactions with application to nicotine dependence. American journal of human genetics.

