# Supplementary material for: Plasma MCP-1 and Cognitive Decline in Patients with Alzheimer’s Disease and Mild Cognitive Impairment: A Two-year Follow-up Study
Source: Sci Rep. 2018 Jan 19;8:1280. doi: 10.1038/s41598-018-19807-y (PMC5775300; doi:10.1038/s41598-018-19807-y)
Supplement: Supplementary file 1 — Supplementary Table 1,2,3 [file 41598_2018_19807_MOESM1_ESM.doc]

**Plasma MCP-1 and Cognitive Decline in Patients with Alzheimer’s Disease and Mild Cognitive Impairment: A Two-year Follow-up Study**

Wei-Ju Lee, Yi-Chu Liao, Yen-Feng Wang, I-Feng Lin, Shuu-Jiun Wang, Jong-Ling Fuh

**Supplementary Table 1**. Summary of logistic regression about the factors predicting cognitive decliner in AD and MCI patients.

|  | B | SE | OR (95 CI%) | P value |
| --- | --- | --- | --- | --- |
| Male (vs. female) | 0.16 | 0.27 | 1.17 (0.69, 2.00) | 0.56 |
| MCP-1 level | 1.91 | 0.81 | 6.77 (1.40, 32.85) | 0.02 |
| APOE4 carrier (vs. non-carrier) | 0.53 | 0.26 | 1.70 (1.03, 2.81) | 0.04 |
| Age | -0.03 | 0.02 | 0.98 (0.94, 1.02) | 0.27 |
| Years of education | -0.01 | 0.03 | 1.00 (0.94, 1.06) | 0.88 |
| CCI | 0.12 | 0.15 | 1.13 (0.83, 1.52) | 0.44 |

MCP-1, Monocyte chemoattractant protein-1; APOE, apolipoprotein E; CCI, Charlson comorbidity index

**Supplementary Table 2. Best gene-gene interaction models identified by GMDR for predicting dementia risk.**

| Best Model | Training balanced accuracy | Testing balanced accuracy | p value |
| --- | --- | --- | --- |
| CCL2 rs1024611 | 0.5463 | 0.5474 | 0.054 |
| CCL2 rs1024611, APOE ε4 | 0.539 | 0.5506 | 0.106 |
| CCR2 rs1799864, CCL2 rs1024611, ApoE ε4 | 0.5267 | 0.5492 | 0.202 |

GMDR, generalized multifactor dimensionality reduction; CCL2, Chemokine ligand 2; CCR2, CC-chemokine receptor 2

**Supplementary Table 3. Best gene-gene interaction models identified by GMDR for predicting plasma MCP-1 level.**

| Best Model | Training balanced accuracy | Testing balanced accuracy | p value |
| --- | --- | --- | --- |
| CCR2 rs1799864 | 0.516 | 0.545 | 0.27 |
| CCR2 rs1799864, CCL2 rs1024611 | 0.495 | 0.567 | 0.57 |
| CCR2 rs1799864, CCL2 rs1024611, APOE ε4 | 0.501 | 0.567 | 0.48 |

GMDR, generalized multifactor dimensionality reduction; MCP-1, Monocyte chemoattractant protein-1; CCR2, CC-chemokine receptor 2; CCL2, Chemokine ligand 2
